# Improved Non-Invasive Preimplantation Genetic Testing for Beta-Thalassemia Using Spent Embryo Culture Medium Containing Blastocoelic Fluid

**DOI:** 10.3389/fendo.2021.793821

**Published:** 2022-01-20

**Authors:** Zhanhui Ou, Yu Deng, Yunhao Liang, Zhiheng Chen, Ling Sun

**Affiliations:** Center of Reproductive Medicine, Guangzhou Women and Children’s Medical Center, Guangzhou Medical University, Guangzhou, China

**Keywords:** preimplantation genetic testing, non-invasive, blastocyst, spent culture medium, β-thalassemia

## Abstract

**Objectives:**

To compare successful beta-thalassemia (β-thalassemia) detection rates obtained using spent culture medium and spent culture medium containing blastocoelic fluid (BF).

**Method:**

This study involved data from 10 couples who underwent preimplantation genetic testing (PGT) for β-thalassemia. A total of 26 samples of spent culture medium containing BF (group A) and 33 samples without BF (group B) were collected and analyzed. The DNA concentration and β-thalassemia detection rates were evaluated.

**Results:**

The *HBB* mutation analysis results of 34 samples were concordant with the biopsy results (34/59, 57.6%). In group A, the *HBB* mutation analysis results of 19 of 26 samples (73.1%) were concordant with the biopsy results. The concordance rate in group A was higher than that in group B (15/33, 45.5%; P < 0.05). The haplotyping results of 38 samples were concordant with the biopsy results (38/59, 64.4%). The concordance rate in group B was 17/33 (51.5%), which was significantly lower than that in group A (21/26, 80.8%) (P < 0.05). In group A, the mean DNA concentration of samples with <10% fragmentation was 107.3 ± 70.1 ng/μL, which was lower than that of samples with ≥10% fragmentation (194.6 ± 28.0 ng/μL) (P < 0.05). However, the detection rates of <10% and ≥10% fragmentation were not significantly different (P > 0.05).

**Conclusion:**

The β-thalassemia detection rate with non-invasive PGT using the spent culture medium containing BF was higher than that using the spent culture medium alone. Fragmentation is associated with DNA concentration in the spent culture medium containing BF.

## Introduction

Beta-thalassemia (β-thalassemia) is one of the most common genetic diseases and is mainly caused by point mutations or small deletions in the beta-globin gene (*HBB*) (1). The prevalence of carriers of β-thalassemia is 2.54% in southern China; for decades, this disease has threatened the lives of millions of people (2). The absence or quantitative reduction of beta-globin chain synthesis leads to moderate or severe anemia (3, 4). Patients with severe β-thalassemia eventually exhibit a high mortality rate, resulting in high global economic and healthcare burdens (5).

Currently, hematopoietic stem cell transplantation is the only definitive cure available for patients with severe forms of β-thalassemia (6, 7). However, this treatment is limited by the small number of HLA-matched healthy donors for most patients (8). Therefore, identification of couples who are β-thalassemia carriers is important to prevent the inheritance of β-thalassemia by their child. The identification methods include prenatal diagnosis and preimplantation genetic testing for monogenic disease (PGT-M). However, prenatal diagnosis may help opt for an abortion if the developing embryo has a problem. PGT-M involves the selection of an embryo without a known genetic disease for transfer during IVF treatment. This has become a routine procedure in in-vitro fertilization clinics worldwide for patients with monogenic diseases.

Biopsy and the subsequent analysis are two key steps in PGT. Currently, blastomere and trophectoderm (TE) biopsies are the most commonly used methods in clinics. However, animal studies have suggested that blastomere biopsy may cause aberrant epigenetic modifications, neurodegenerative disorders, and ovary dysfunction in offspring (9–12). Additionally, TE biopsy involves the removal of cells that are destined to form the placenta, and a previous study showed that TE quality may affect the implantation potential (13). Importantly, a biopsy of a large amount of TE may affect pregnancy rates (14). These results suggest that embryo biopsy can injure embryos and affect fetal development. This procedure is also time- and labor-intensive.

To overcome the limitations associated with biopsy, some studies have focused on non-invasive PGT using cell-free DNA present in the spent embryo culture medium (15–17). However, the small quantity of DNA present in the embryo culture medium is not sufficient to generate accurate non-invasive PGT results. Magli et al. (18) reported that blastocoelic fluid (BF), the fluid within the blastocyst, contains a high concentration of DNA. Furthermore, high percentage of fragmentation in the embryo may be increased the high concentration of DNA in the spent culture medium (19). However, to the best of our knowledge, the relationship between the successful detection of monogenic disorders and the rate of fragmentation has not been studied.

Currently, only a few studies have investigated non-invasive PGT-M. Wu et al. (16) reported that alpha-thalassemia-SEA can be successfully diagnosed using quantitative polymerase chain reaction (qPCR) analysis of spent embryo culture medium. However, not all mutations can be detected by qPCR, and this may lead to a misdiagnosis. Thus, the clinical application of this method is limited. Liu et al. (15) reported that non-invasive genetic testing of embryos for *HBB* (IVS-II-654) by next-generation sequencing (NGS) can produce results concordant with those of biopsied cells. However, they used only 10 samples for *HBB* detection and found that the DNA concentration in the media of degenerated or fragmented embryos was slightly higher than that in the media of blastocysts.

In the present study, we performed whole-genome amplification (WGA) using the multiple annealing and looping-based amplification cycle method to amplify cell-free nuclear DNA present in the spent culture medium. The amplified DNA was then analyzed by Sanger sequencing and NGS to detect β-thalassemia. The results were compared to those obtained from biopsied TE cells. Moreover, the results from spent culture medium were compared with those from spent culture medium containing BF. The association between fragmentation and the successful detection rate of β-thalassemia using spent culture medium only and spent culture medium containing BF was determined. This study may further improve non-invasive PGT-M for β-thalassemia detection using spent culture medium.

## Materials and Methods

### Ethical Approval

The study was exempt from ethical approval by the ethics committee of Guangzhou Women and Children’s Medical Center (Guangzhou, China). Written informed consent was obtained from the study participants.

### Patient Cohort and Treatment

Ten couples who were β-thalassemia carriers were recruited from the Reproductive Medicine Centre of the Guangzhou Women and Children’s Medical Center for PGT. Ten couples underwent 11 PGT-M cycles between April 2018 and July 2020. Patients were treated with gonadotropin-releasing hormone antagonists and stimulated with a recombinant follicle-stimulating hormone.

### Embryo Culture and Evaluation

After intracytoplasmic sperm injection, all fertilized oocytes were cultured for 1–3 days in G1 culture medium (Vitrolife, Goteborg, Sweden) with pre-gassed mineral oil. Day 3 embryos were assessed, including the level of fragmentation (a fragment was defined as an extracellular membrane-bound cytoplasmic structure that was <40 mm in diameter) according to the Istanbul consensus (20). On day 3, the embryos were transferred into 25 μL of G2 droplet and cultured individually to form blastocysts. Blastocyst scoring and grading were performed according to the Gardner scoring system (21).

### Biopsy and Sample Collection

Biopsy was performed on day 5 or 6 depending on the blastocyst grade on the day of biopsy (22). Fifty-nine blastocysts from 11 oocyte retrieval cycles were included in this study and were divided into two groups according to the time of retrieval. For group A, oocytes were retrieved between April 2018 and April 2019 and for group B, oocytes were retrieved between May 2019 and July 2020. In group A (n = 26), the blastocysts hatched first with the inner cell mass at approximately the 9 o’clock position, and one or two laser pulses were used to create a small breach (10 mm) in the thinnest zona pellucida (ZP) at approximately the 3 o’clock position. During this process, the laser inevitably hits the TE cells adhered to the ZP, thus releasing the BF into the culture medium, and then blastocysts collapse and the TE cells crumble. Twenty microliters of the culture medium containing BF was collected. After a biopsy of 5–10 TE cells, the blastocysts were cryopreserved by vitrification according to the manufacturer’s protocol (ARSCI, Inc., Longueuil, Canada) and then stored in liquid nitrogen. In group B (n = 33), 20 μL of the culture medium on D5 or D6 was collected, and then the blastocysts were hatched and biopsied as mentioned previously.

**Figure 1:**
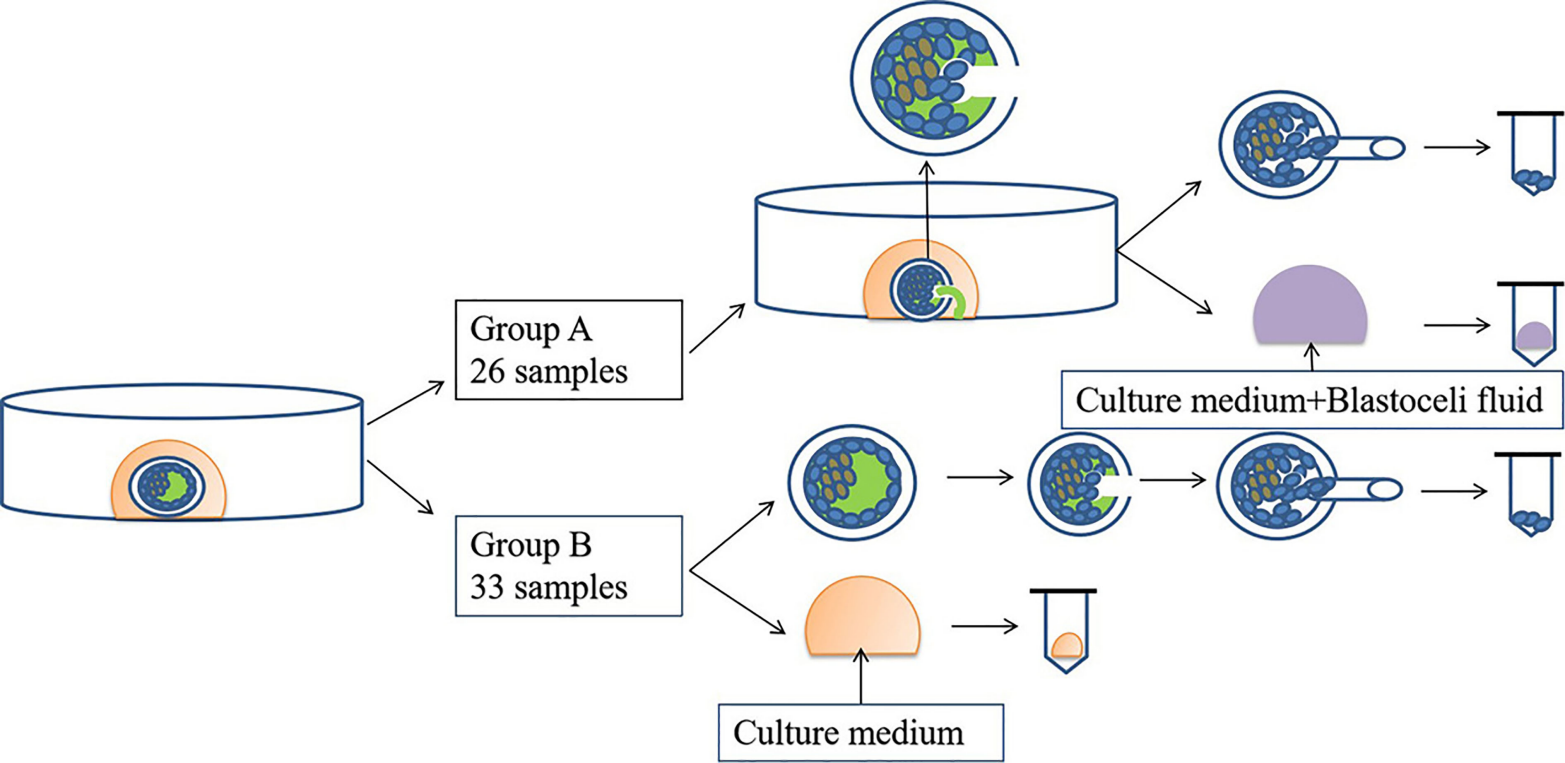
Flow diagram of this study.

To prevent cross-contamination of media, different tips were used for each blastocyst. Each sample was then transferred into RNase–DNase-free PCR tubes containing 5 μL of cell lysis buffer (Yikon Genomics, Shanghai, China). Equal amounts of G2 without blastocyst culture were collected and used as the negative controls. All collected samples were stored at −80°C until use. A flow diagram of this study is shown in **Figure 1**.

### WGA of the Culture Medium and Biopsied Cells

The WGA of DNA in the spent culture medium was performed using the multiple annealing and looping-based amplification cycle methods and a Single Cell Whole-Genome Amplification Kit (Cat. no. KT110700150; Yikon Genomics, China) according to the manufacturer’s standard protocol. Briefly, 10 µL of a mixture of 20 µL of culture medium and 5 µL of lysis buffer was transferred into a PCR tube, and then 1 μL of lysis enzyme was added into the tube. The samples were incubated at 50°C for 50 min, and then at 80°C for 10 min in a thermocycler.

### Single-Nucleotide Polymorphism Analysis by NGS and *HBB* Mutation Detection by Sanger Sequencing

The WGA products were used for *HBB* mutation detection and NGS-based single-nucleotide polymorphism (SNP) haplotyping. Ninety SNP markers located 1 Mb upstream and downstream of *HBB* were chosen for haplotyping. The identified SNP sites were submitted to Ion Ampliseq Designer for primer design. Sequencing libraries were prepared using a gene sequencing library kit (XK-038; Yikon Genomics, China). Libraries approximately 350 base pairs in size were chosen and processed for paired-end high-throughput sequencing on an Illumina Nextseq500 sequencer (San Diego, CA, USA). All procedures were performed according to the manufacturer’s protocol. The sequenced data were aligned and visualized using ChromGo software (http://chromgo.yikongenomics.cn:7000/#/home; Yikon Genomics). Sanger sequencing using the corresponding primers was performed to detect mutations in *HBB*.

### Evaluation of the Results

*HBB* detection was performed in two steps. First, we identified the amplification rate of *HBB* (mutation) and informative SNP site. Second, the accuracy of the *HBB* mutation rate was determined by comparing it with that obtained from TE biopsy.

### Statistical Analysis

Statistical analysis was performed using SPSS software v. 19 for Windows (SPSS, Inc., Chicago, IL, USA) by applying parametric and nonparametric tests as appropriate. Continuous variables are expressed as mean ± standard deviation and analyzed using Student’s *t*-test. Categorical variables are expressed as percentage and analyzed using χ^2^ or Fisher’s exact test depending on the sample size. Differences with P < 0.05 were considered significant.

## Results

### Quantitative Analysis of WGA DNA in Spent Culture Medium

The mean DNA concentration in 26 samples of spent culture medium containing BF (147.6 ± 69.8 ng/μL) was significantly higher than that in 33 samples of spent culture medium only (40.9 ± 31.7 ng/μL) (P < 0.05; **Table 1**).

**Table 1:** Samples from spent culture medium evaluated by WGA.

	Spent culture medium contained BF	Spent culture medium only	P
DNA concentration (ng/μL)	147.6 ± 69.8	41.5 ± 31.7	0.000
SNP sites	37.5 ± 15.1	19.8 ± 17.4	0.000
Informative SNP sites	13.2 ± 6.4	6.3 ± 5.9	0.000
Successfully detection rate (%) by *HBB* mutation	73.1 (19/26)	45.5 (15/33)	0.033
Successfully detection rate (%) by haplotype analyze and *HBB* mutation	80.8% (21/26)	51.5% (17/33)	0.019

### Association Between Fragmentation and DNA Concentration

Most embryos (57/59) exhibited less than 20% fragmentation on day 3 (**Table 2** and **Supplementary Tables 1, 2**). Therefore, we divided the embryos into two groups according to the level of fragmentation (<10% or ≥10%). In the samples of spent culture medium containing BF, the mean DNA concentration of samples with <10% fragmentation was 107.3 ± 70.1 ng/μL, which was lower than that of samples with ≥10% fragmentation at 194.6 ± 28.0 ng/μL (P < 0.05; **Table 2**). In the samples of spent culture medium only, the mean DNA concentration in samples with <10% fragmentation was 42.2 ± 34.7 ng/μL compared with 39.4 ± 28.8 ng/μL in samples with ≥10% fragmentation, showing no significant difference (P > 0.05; **Table 2**).

**Table 2:** Analysis of fragmentation and DNA concentration and successful detection rate.

	Fragmentation <10%	Fragmentation ≥10%	P
DNA concentration in group A (ng/μL)	107.3 ± 70.1	194.6 ± 28.0	0.000
Successful detection rate analyzed with *HBB* in group A	78.5% (11/14)	66.7 (8/12)	0.495
Successful detection rate analyzed with haplotype and *HBB* mutation in group A	78.6% (11/14)	83.3% (10/12)	0.848
DNA concentration in group B (ng/μL)	42.2 ± 34.7	39.4 ± 28.8	0.804
Successful detection rate analyzed with *HBB* in group B	38.9% (7/18)	53.3% (8/15)	0.406
Successful detection rate analyzed with haplotype and *HBB* mutation in group B	50.0% (9/18)	53.5% (8/15)	0.849

### *HBB* Mutation Detection

For each sample, *HBB* was sequenced using the corresponding primers synthesized on the basis of the *HBB* mutation. For all 59 TE biopsies, conclusive results were obtained by NGS and Sanger sequencing. In the spent culture medium, amplification of 34 samples was successful, whereas that of 25 samples was not successful. The results of 34 samples with successful amplification were concordant with the results of biopsy (34/59, 57.6%). In group A, the amplification of 19 of 26 samples (73.1%) was successful, and the results of all of them were concordant with those of biopsy. The concordance rate in group B was 15/33 (45.5%), which is lower than that in group A (P < 0.05; **Table 1**, **Supplementary Tables 1, 2**).

### *HBB* SNP Linkage Analysis

Ninety SNP sites located 1 Mb upstream and downstream of *HBB* were selected for SNP linkage analysis. The number of sites in group A (37.5 ± 15.1) was higher than that in group B (19.8 ± 17.4; P < 0.05; **Table 1**, **Supplement Table 1, 2**). To further analyze the results, the number of informative SNP sites between groups A and B was compared. Group A samples had 13.2 ± 6.4 informative SNP sites, which were higher than those of group B samples (6.3 ± 5.9 informative SNP sites) (P < 0.05; **Table 1**, **Supplementary Tables 1, 2**).

At least four informative SNPs (two flanking markers of *HBB* mutation) were used in the linkage analysis. By SNP linkage analysis using informative SNP sites, 4 more samples were diagnosed successfully. In group A, the diagnosis rate was up to 80.8% (21/26), and all the diagnosis results were concordant with the results of biopsy. The corresponding rate was 51.5% (17/33) in group B, which was lower than that in group A (P < 0.05; **Table 1**, **Supplementary Tables 1, 2**).

### Association Between Fragmentation and the Rate of Successful B-Thalassemia Detection

In the samples of spent culture medium containing BF, the successful detection rate of *HBB* mutations in samples with <10% fragmentation (78.5%, 11/14) was not significantly different from that of samples with ≥10% fragmentation (66.7%, 8/12) (P > 0.05; **Table 2**); no significant difference was observed in the analysis of the SNP sites (78.6% (11/14) vs. 83.3(10/12), P > 0.05; **Table 2**). In the samples of spent culture medium only, the successful detection rate of these two groups was 38.9% (7/18) and 53.3% (8/15), respectively, which was not significantly different (P > 0.05; **Table 2**), and no significant difference was observed after in the analysis of the SNP sites (50.0% (9/18) vs. 53.3% (8/15), P > 0.05; **Table 2**).

## Discussion

Non-invasive chromosome screening of human blastocysts based on the sequencing of genomic DNA in the culture medium has been increasingly performed, with acceptable results, but it has not been used in clinical settings (17, 23, 24). The main reason for this could be its diagnosis accuracy. Xu et al. (17) performed NGS of the spent culture medium and obtained ploidy information of all 24 chromosomes. By comparing each result with that of the corresponding whole donated embryo, a correlation analysis was observed to identify chromosomal abnormalities (sensitivity 0.882 and specificity 0.840). Yin et al. (24) reported that 78.67% (59/75) of the NGS results obtained from the spent culture medium were interpretable, but had low sensitivity and diagnostic accuracy.

However, only a few studies have used spent culture medium for non-invasive PGT-M (15, 16, 25). Liu et al. (15) used spent culture media to diagnose the β-thalassemia IVS-II-654 mutation by NGS. The results of seven samples of culture medium (7/10, 70%) were concordant with the corresponding embryo biopsy results. They analyzed 10 samples of culture medium, and eight of them were originally from arrested or degenerated embryos and two were blastocysts. This indicated that most embryos were fragmented, which may have affected the results.

To further evaluate the use of this method in the clinical setting, all samples of the culture medium used in the present study were from blastocysts that were more suitable for transfer. Besides, we compared the successful β-thalassemia detection rates using spent culture medium and spent culture medium containing BF. In addition, we evaluated the association between fragmentation and β-thalassemia detection rates.

The results showed that the DNA concentration and detection rates, *HBB* SNP linkage sites, and informative SNP linkage sites for the spent culture medium containing BF were significantly higher than those for the spent culture medium. This may be because of the DNA present in the spent culture medium containing BF and fragmentation of apoptotic cells released into the culture medium after zona breaching with the laser (19). Furthermore, extremely low DNA concentrations, less than the sensitivity of WGA, might have led to the failure of WGA in the spent culture medium only (15). Clinically, blastocysts undergo zona breaching with the laser and collapse before vitrification or biopsy; thus, an additional process is not required in the clinic settings. This manipulation causes the expulsion of BF into the culture medium. Therefore, the spent culture medium containing BF produced better results than the spent culture medium in non-invasive PGT-M. This type of culture medium may be the best option for non-invasive PGT-M.

Only a few studies have confirmed a relationship between fragmentation and the spent culture medium (15, 24). To further validate the role of cell fragmentation in the spent culture medium for β-thalassemia detection, the association between fragmentation and DNA concentration was evaluated. We detected only two embryos with more than 20% fragmentation on day 3. This is because extensive fragmentation in embryos is associated with reduced embryo quality and failure to develop into blastocysts (26, 27). Therefore, we divided the embryos into two groups according to the fragmentation level (<10% and ≥10%). In the samples of spent culture medium containing BF, the DNA concentration in the samples with <10% fragmentation was significantly lower than that in the samples with ≥10% fragmentation; however, no significant difference was observed in the spent culture medium only (without assisted hatching). A possible explanation is the fragmentation from apoptotic cells released into the culture medium after zona breaching, and the level of fragmentation may be associated with the DNA concentration.

We also evaluated the association between fragmentation and the successful detection rate of *HBB* mutations. The results showed that the successful detection rate of *HBB* mutations did not significantly differ in the spent culture medium containing BF and spent culture medium only group, regardless of the fragmentation level. A reason for this result may be the poor quality of highly degraded source DNA affecting the successful detection rate (28). Successful detection with DNA present in spent culture medium depends on the presence of sufficient concentrations of high-quality DNA. Human embryos can self-correct by eliminating abnormal blastomeres as cell debris (29). This may affect the quality of source DNA.

We also analyzed the *HBB* SNP linkage sites to reduce the risk of unacceptable misdiagnosis results. The European Society of Human Reproduction and Embryology recommends including at least two flanking markers while detecting indirect mutations (22). The detection rate of *HBB* mutations using the culture medium containing BF was 73.1%. However, the detection rate was 80.8% when the informative SNP linkage sites were analyzed. As the successful detection rate of the *HBB* inheritance state increased for 4 samples by including these two upstream and two downstream markers, we recommend using the *HBB* SNP linkage sites and mutation by NGS to increase the robustness and sensitivity of the test. The results of informative SNP linkage sites were better than those of *HBB* mutation detection only, and this might be because of the fragmented DNA template in the culture medium and because the primers could not amplify the target area. However, the use of more SNP linkage sites might improve the robustness of amplification by reducing the effect of recombination. However, even with this method, conclusive results may still not be obtained for some samples because the DNA concentration might be below the detection sensitivity of WGA, leading to amplification failure.

Considering the low concordance rate with invasive PGT-M results, these methods currently cannot fully replace the use of invasive TE biopsy for PGT-M. To increase the successful detection rate of β-thalassemia mutations using spent culture medium, several methods should be improved. First, the culture medium volume should be optimized to obtain high DNA concentrations without affecting embryo growth. Second, the sensitivity of WGA to amplify DNA present at low levels in the culture medium and sensitivity of NGS to detect low levels of DNA template should be improved. Third, NGS primers should detect the *HBB* SNP linkage sites and *HBB* mutation to reduce the risk of misdiagnosis. Fourth, measures against maternal-cell contamination should be taken, such as fertilization using intracytoplasmic sperm injection and removing cumulus cells (to the maximum extent possible). Fifth, embryos should be cultured until day 5 or 6 in the two-step culture medium. Finally, spent culture medium after zona breaching may be more suitable for non-invasive PGT-M.

There were some limitations to the present study. First, there were relatively few, only 59, embryos available for analysis. Second, the use of 10% fragmentation as the cutoff value may not have been optimal. Selecting a higher level of fragmentation to evaluate associations may improve the results. Finally, the NGS primers did not include the *HBB* mutations.

In conclusion, we found that (1) the spent culture medium containing BF was better for detecting β-thalassemia in non-invasive PGT than the spent culture medium only (2); detecting the HBB SNP linkage sites and mutation can increase the detection sensitivity; and (3) the fragmentation is associated with the DNA concentration in the spent culture medium containing BF. However, the successful detection rate requires further improvements, and at present, PGT-M with cell-free DNA in spent culture medium should not be applied in routine clinical settings for diagnostic purposes.

## Ethics Statement

The studies involving human participants were reviewed and approved by the ethics committee of Guangzhou Women and Children’s Medical Center. The patients/participants provided their written informed consent to participate in this study.

## Author Contributions

ZHO and LS—conception and design of the study. ZHO and YHL—performed all the molecular genetic analyses and participated in the design of the study. ZHO, YD and ZHC—data collection, statistical analysis, construction of figures and tables. ZHO, ZHC and YD—drafted the article. All— reviewed the manuscript and approved the version to be published.

## Funding

This study was supported by Health Science and technology project of Guangzhou (20201A011029 and 20211A011026), and Youth medicine research and development program for Reproductive Medicine from Chinese Medical Association (17020150684).

## Conflict of Interest

The authors declare that the research was conducted in the absence of any commercial or financial relationships that could be construed as a potential conflict of interest.

## Publisher’s Note

All claims expressed in this article are solely those of the authors and do not necessarily represent those of their affiliated organizations, or those of the publisher, the editors and the reviewers. Any product that may be evaluated in this article, or claim that may be made by its manufacturer, is not guaranteed or endorsed by the publisher.
