# Supplementary material for: Improved Non-Invasive Preimplantation Genetic Testing for Beta-Thalassemia Using Spent Embryo Culture Medium Containing Blastocoelic Fluid
Source: Front Endocrinol (Lausanne). 2022 Jan 20;12:793821. doi: 10.3389/fendo.2021.793821 (PMC8810495; doi:10.3389/fendo.2021.793821)
Supplement: Supplementary file 1 [file DataSheet_1.docx]

Supplement table 1. Results of spent culture medium containing BF.

| Sample | Father mutation | Mother mutation | Fragmentation in D3 embryo | Blastocyst quality | SNP site | Informative SNP site ^#^ | HBB mutation detection | HBB mutation detection and haplotype analyze | TE results |
| --- | --- | --- | --- | --- | --- | --- | --- | --- | --- |
| 1 | CD 41/42 | CD 41/42 | 0 | ⅤCB | 48 | 13 (8+5) | Homozygous | Homozygous | Homozygous |
| 2 | -28 | CD 17 | 0 | ⅤBC | 34 | 17 (9+8) | Compound heterozygous | Compound heterozygous | Compound heterozygous |
| 3 | -28 | CD 17 | 0 | ⅣCB | 33 | 18 (10+8) | Wild-type | Wild-type | Wild-type |
| 4 | -28 | CD 17 | 0 | ⅤCB | 17 | 9 (4+5) | Compound heterozygous | Compound heterozygous | Compound heterozygous |
| 5 | -28 | CD 17 | 0 | ⅣCB | 14 | 2 (2+0) | Wild-type | Wild-type | Wild-type |
| 6 | -28 | CD 41/42 | 0 | ⅤBA | 11 | 5 (3+2) | Fail | Fail | Wild-type |
| 7 | -28 | CD 41/42 | 0 | ⅤBB | 12 | 8 (5+3) | Fail | Fail | Heterozygous |
| 8 | -28 | CD 41/42 | 0 | ⅤBB | 6 | 2 (1+1) | Fail | Fail | Wild-type |
| 9 | -28 | CD 41/42 | 0 | ⅤAA | 46 | 21 (12+9) | Compound heterozygous | Compound heterozygous | Compound heterozygous |
| 10 | -28 | CD 41/42 | 0 | ⅤBB | 4 | 21 (12+9) | Wild-type | Wild-type | Wild-type |
| 11 | -28 | CD 41/42 | 0 | ⅤCB | 46 | 24 (14+10) | Wild-type | Wild-type | Wild-type |
| 12 | -28 | CD 41/42 | 5 | ⅤBC | 44 | 24 (14+10) | Wild-type | Wild-type | Wild-type |
| 13 | IVS-II-654 | IVS-II-654 | 5 | ⅤBB | 47 | 15 (9+6) | Heterozygous | Heterozygous | Heterozygous |
| 14 | IVS-II-654 | IVS-II-654 | 5 | ⅤCB | 42 | 14 (9+5) | Wild-type | Wild-type | Wild-type |
| 15 | IVS-II-654 | IVS-II-654 | 10 | ⅤCB | 18 | 1 (0+1) | Fail | Fail | Wild-type |
| 16 | IVS-II-654 | IVS-II-654 | 10 | ⅣAB | 27 | 4 (3+1) | Fail | Fail | Homozygous |
| 17 | IVS-II-654 | IVS-II-654 | 10 | ⅣBB | 50 | 14 (9+5) | Heterozygous | Heterozygous | Heterozygous |
| 18 | IVS-II-654 | IVS-II-654 | 10 | ⅤBB | 48 | 15 (11+4) | Fail | Wild-type | Wild-type |
| 19 | IVS-II-654 | IVS-II-654 | 10 | ⅤCB | 51 | 15 (11+4) | Fail | Heterozygous | Heterozygous |
| 20 | IVS-II-654 | IVS-II-654 | 10 | ⅤCB | 50 | 15 (11+4) | Wild-type | Wild-type | Wild-type |
| 21 | IVS-II-654 | IVS-II-654 | 10 | ⅤCB | 55 | 15 (10+5) | Homozygous | Homozygous | Homozygous |
| 22 | IVS-II-654 | IVS-II-654 | 15 | ⅣCB | 37 | 14 (10+4) | Heterozygous | Heterozygous | Heterozygous |
| 23 | IVS-II-654 | IVS-II-654 | 15 | ⅣCB | 42 | 13 (9+4) | Heterozygous | Heterozygous | Heterozygous |
| 24 | IVS-II-654 | IVS-II-654 | 15 | ⅣCB | 51 | 15 (9+6) | Heterozygous | Heterozygous | Heterozygous |
| 25 | IVS-II-654 | IVS-II-654 | 15 | ⅣBC | 50 | 15 (8+7) | Heterozygous | Heterozygous | Heterozygous |
| 26 | IVS-II-654 | IVS-II-654 | 40 | ⅢCB | 50 | 15 (9+6) | Wild-type | Wild-type | Wild-type |

^#^ Number in the brackets were informative SNP site for father’s mutation on the left and informative SNP site for mother’s mutation on the right.

Supplement table 2. Results of spent culture medium only.

| Sample | Paternal mutation | Maternal mutation | Fragmentation in D3 embryo | Blastocyst  quality | SNP site | Informative SNP site ^#^ | HBB mutation detection | HBB mutation detection and haplotype analyze | TE results |
| --- | --- | --- | --- | --- | --- | --- | --- | --- | --- |
| 1 | IVS-II-654 | IVS-II-654 | 0 | ⅤBB | 20 | 8 (4+4) | Homozygous | Homozygous | Homozygous |
| 2 | IVS-II-654 | IVS-II-654 | 0 | ⅤBB | 2 | 1 (1+0) | Fail | Fail | Heterozygous |
| 3 | IVS-II-654 | CD 41/42 | 0 | ⅤAA | 10 | 0 (0+0) | Fail | Fail | Compound heterozygous |
| 4 | IVS-II-654 | CD 41/42 | 0 | ⅤBA | 9 | 2 (1+1) | Heterozygous | Heterozygous | Heterozygous |
| 5 | IVS-II-654 | CD 41/42 | 0 | ⅤBA | 11 | 2 (1+1) | Fail | Fail | Heterozygous |
| 6 | CD 41/42 | CD 41/42 | 0 | ⅤCB | 3 | 0 (0+0) | Fail | Fail | Heterozygous |
| 7 | CD 41/42 | IVS-II-654 | 0 | ⅣBC | 9 | 3 (1+2) | Fail | Fail | Heterozygous |
| 8 | IVS-II-654 | CD 71-72 | 5 | ⅤBA | 34 | 11 (7+4) | Fail | Heterozygous | Heterozygous |
| 9 | IVS-II-654 | CD 71-72 | 5 | ⅤBA | 20 | 7 (4+3) | Fail | Fail | Heterozygous |
| 10 | IVS-II-654 | CD 71-72 | 5 | ⅤBB | 57 | 20 (11+9) | Fail | Wild-type | Wild-type |
| 11 | IVS-II-654 | CD 71-72 | 5 | ⅤBB | 22 | 7 (3+4) | Fail | Fail | Wild-type |
| 12 | IVS-II-654 | CD 71-72 | 5 | ⅤBB | 57 | 17 (10+7) | Heterozygous | Heterozygous | Heterozygous |
| 13 | IVS-II-654 | CD 71-72 | 5 | ⅤBB | 52 | 14 (6+8) | Compound heterozygous | Compound heterozygous | Compound heterozygous |
| 14 | IVS-II-654 | CD 71-72 | 5 | ⅥCB | 37 | 13 (8+5) | Compound heterozygous | Compound heterozygous | Compound heterozygous |
| 15 | IVS-II-654 | CD 41/42 | 5 | ⅤBA | 20 | 3 (1+2) | Heterozygous | Heterozygous | Heterozygous |
| 16 | CD 41/42 | CD 41/42 | 5 | ⅤAB | 35 | 15 (4+11) | Compound heterozygous | Compound heterozygous | Compound heterozygous |
| 17 | CD 41/42 | CD 41/42 | 5 | ⅤCB | 3 | 0 (0+0) | Fail | Fail | Heterozygous |
| 18 | CD 41/42 | CD 41/42 | 5 | ⅤCB | 4 | 2 (0+2) | Fail | Fail | Heterozygous |
| 19 | IVS-II-654 | CD 71-72 | 10 | ⅤBC | 16 | 6 (3+3) | Fail | Fail | Compound heterozygous |
| 20 | IVS-II-654 | IVS-II-654 | 10 | ⅤBA | 52 | 17 (7+10) | Wild-type | Wild-type | Wild-type |
| 21 | IVS-II-654 | IVS-II-654 | 10 | ⅤBB | 1 | 0 (0+0) | Fail | Fail | Wild-type |
| 22 | IVS-II-654 | IVS-II-654 | 10 | ⅤCB | 33 | 10 (6+4) | Heterozygous | Heterozygous | Heterozygous |
| 23 | IVS-II-654 | IVS-II-654 | 10 | ⅤBC | 18 | 5 (3+2) | Fail | Fail | Homozygous |
| 24 | IVS-II-654 | CD 41/42 | 10 | ⅤCB | 27 | 8 (5+3) | Wild-type | Wild-type | Wild-type |
| 25 | CD 41/42 | CD 41/42 | 10 | ⅣCB | 6 | 1 (1+0) | Fail | Fail | Heterozygous |
| 26 | CD 41/42 | CD 41/42 | 10 | ⅣCB | 22 | 8 (4+4) | Heterozygous | Heterozygous | Heterozygous |
| 27 | IVS-II-654 | IVS-II-654 | 15 | ⅤBB | 8 | 3 (2+1) | Heterozygous | Heterozygous | Heterozygous |
| 28 | IVS-II-654 | IVS-II-654 | 15 | ⅤBB | 15 | 3 (1+2) | Fail | Fail | Heterozygous |
| 29 | IVS-II-654 | IVS-II-654 | 15 | ⅤBC | 44 | 13 (11+2) | Heterozygous | Heterozygous | Heterozygous |
| 30 | IVS-II-654 | CD 41/42 | 15 | ⅣBC | 2 | 0 (0+0) | Fail | Fail | Heterozygous |
| 31 | -28 | -28 | 15 | ⅢAC | 4 | 0 (0+0) | Fail | Fail | Wild-type |
| 32 | -28 | -28 | 30 | ⅣBC | 5 | 1 (0+1) | Homozygous | Homozygous | Homozygous |
| 33 | CD 41/42 | IVS-II-654 | 15 | ⅣBC | 14 | 3 (1+2) | Heterozygous | Heterozygous | Heterozygous |

^#^ Number in the brackets were informative SNP site for father’s mutation on the left and informative SNP site for mother’s mutation on the right.
